# Evidence of Local Persistence of Human Anthrax in the Country of Georgia Associated with Environmental and Anthropogenic Factors

**DOI:** 10.1371/journal.pntd.0002388

**Published:** 2013-09-05

**Authors:** Ian T. Kracalik, Lile Malania, Nikoloz Tsertsvadze, Julietta Manvelyan, Lela Bakanidze, Paata Imnadze, Shota Tsanava, Jason K. Blackburn

**Affiliations:** 1 Spatial Epidemiology and Ecology Research Lab, Department of Geography, University of Florida, Gainesville, Florida, United States of America; 2 Emerging Pathogens Institute, University of Florida, Gainesville, Florida, United States of America; 3 National Center for Disease Control and Public Health, Tbilisi, Georgia; 4 Agrarian University of Georgia, Tbilisi, Georgia; Oxford University Clinical Research Unit, Viet Nam

## Abstract

**Background:**

Anthrax is a soil-borne disease caused by the bacterium *Bacillus anthracis* and is considered a neglected zoonosis. In the country of Georgia, recent reports have indicated an increase in the incidence of human anthrax. Identifying sub-national areas of increased risk may help direct appropriate public health control measures. The purpose of this study was to evaluate the spatial distribution of human anthrax and identify environmental/anthropogenic factors associated with persistent clusters.

**Methods/Findings:**

A database of human cutaneous anthrax in Georgia during the period 2000–2009 was constructed using a geographic information system (GIS) with case data recorded to the community location. The spatial scan statistic was used to identify persistence of human cutaneous anthrax. Risk factors related to clusters of persistence were modeled using a multivariate logistic regression. Areas of persistence were identified in the southeastern part of the country. Results indicated that the persistence of human cutaneous anthrax showed a strong positive association with soil pH and urban areas.

**Conclusions/Significance:**

Anthrax represents a persistent threat to public and veterinary health in Georgia. The findings here showed that the local level heterogeneity in the persistence of human cutaneous anthrax necessitates directed interventions to mitigate the disease. High risk areas identified in this study can be targeted for public health control measures such as farmer education and livestock vaccination campaigns.

## Author Summary

Anthrax is a zoonotic bacterial disease that occurs nearly worldwide. Despite a large number of countries reporting endemic anthrax, persistence of the disease appears to be associated with specific ecological factors related to soil composition and climatic conditions. Human cases are most often associated with handling infected livestock or contaminated meat and most cases are in cutaneous form (skin infections). Following the collapse of the Soviet Union, the country of Georgia has undergone major restructuring in land management and livestock handling and anthrax remains a serious public health risk. Few studies have evaluated the local spatial patterns of human anthrax. Here we identify areas on the landscape where human cutaneous anthrax persisted over the last decade. Persistence was found to be associated with both anthropogenic and environmental factors including soil pH and livestock density. These findings aid in the establishment of spatial baseline estimates of the disease and allow public health officials to adopt targeted anthrax control strategies, such as livestock vaccination campaigns and farmer education.

## Introduction

Recently it has been suggested that the burden of anthrax has not been fully realized [1]. Interest in anthrax as a biological weapon has grown since the 2001 bioterrorist attacks [2] yet the disease is often undervalued as a public health threat. Anthrax is considered a neglected zoonosis, disproportionately afflicting rural areas in developing nations [3]. Although several countries have implemented successful control strategies, the disease continues to persist globally with an estimated 20,000 to 100,000 new cases yearly [4]. However, the true disease burden is likely unknown. Areas most heavily impacted by the disease include countries of sub-Saharan Africa, and former Soviet states in Southwestern and Central Asia [5], [6].

The causative agent, *Bacillus anthracis*, is a soil-borne, Gram-positive bacterium, which primarily infects herbivores and secondarily afflicts humans. The bacterium is able to persist in the environment for years, possibly even decades, in alkaline soils with high calcium and organic matter [7] and has been shown to be limited geographically by other environmental factors such as temperature [8], [9], [10]. Human transmission is often a result of coming into contact with infected animals or contaminated animal materials during agricultural activities including the butchering of livestock or industrial exposures through the processing of hair and bone [8], [9], [10]. Manifestation of the disease in humans typically occurs in three acute clinical forms with cutaneous anthrax comprising ∼95% of all reported cases, followed by gastrointestinal and inhalation anthrax [8], [9], [10]. Although the most common form of the disease is treatable with antimicrobials, if left untreated, human cutaneous anthrax can be highly fatal with case fatality ratios in excess of 20% [11], [12]. In neglected endemic regions anthrax can result in substantial economic losses from livestock mortality and lost worker productivity.

In the country of Georgia, anthrax is classified as endemic [13] and has persisted for centuries with the first description of human disease thought to be anthrax documented in 1697 [4]. Following the collapse of the Soviet Union, public and veterinary health services in Georgia experienced tremendous setbacks due to deteriorating organization and finances [14]. During this period of transition to independence Georgia experienced an increase in the number of reported human anthrax cases with 118 reported cases during 1991 to 1996 compared to 36 cases reported during 1985 to 1990 [15]. Recent evidence indicates that the situation has continued to worsen and that incidence rates have surpassed that of neighboring countries, including hyperendemic Turkey [5]. Although human anthrax outbreaks are in some instances sporadic, as evident by the recent outbreak in Bangladesh after a more than 20 year absence [14], [16], the ability of the bacterium to survive in the environment [16], [17] can give rise to areas of persistence and disease recurrence. Additionally, the heterogeneous nature of the disease [18] necessitates control efforts that target high risk areas rather than employing blanket control efforts. This is especially true in developing nations where resources are often limited. Therefore, identifying local areas of persistent may allow for the more efficient implementation of public health intervention strategies such as livestock vaccination, increased awareness of the disease, and placement of improved diagnostics for local veterinary and human health care facilities.

This study had three objectives: 1) to analyze human anthrax at a local scale to better understand the distribution and burden of disease over the past decade, 2) identify areas of persistence for targeted public health control measures 3) identify environmental and anthropogenic factors associated with areas of persistence.

## Methods

### Ethics Statement

No human subjects work was undertaken in this study, human anthrax case data were extracted from government reports prepared and approved by the Georgian National Center for Disease Control and Public Health (NCDC). These government reports provided summarized count data of patients diagnosed at health care facilities by category of disease and year. All data were anonymised.

### Data Collection and Management

Anthrax is a nationally reportable infectious disease in Georgia. Surveillance and documentation within the country is undertaken by the NCDC, which is comprised of a reporting network of 11 regional and 66 district public health centers [7], [19]. A spatial database of reported human cutaneous anthrax was constructed with a geographic information system (GIS) using case data from 2000 to 2009 (Figure S1). Latitude and longitude coordinate pairs were matched to the reported community using the National Geospatial Intelligence Agency GEOnet names server [20], Index Mundi [21], and the GeoNames database [22]. Population data for each community were derived from the Population Statistics of Eastern Europe database [23] for the year 2002. Of the communities that were included in this human anthrax database we were unable to identify population estimates for seven of these locations. In these instances, the population of these seven communities were estimated using the Gridded Population of the World (GPW3) population dataset for the year 2000 [24]. Yearly population estimates were extrapolated using the United Nations (UN) medium variant population growth rates for Georgia during the periods 2000 to 2005 (−1.17) and 2005 to 2010 (−0.57) (http://data.un.org/) using the following formula:



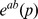
where *a* is the UN population growth rate for either the period 2000 to 2005 or 2005 to 2010 aforementioned, *b* is the number of years from the reference population and *(p)* is the population.

### Epidemiological Analysis

Yearly national incidence rates per one million population were calculated with the total number of cases reported in Georgia for each year as the numerator and the yearly national population obtained from GeoStat [25] as the denominator. The study period was divided into two equal five-year periods to identify potential changes in the reporting of the disease over time and space. Cumulative incidence risk was calculated per community for each five-year period (2000 to 2004) and (2005 to 2009) with the total number of cases during each period as the numerator and the median year community population of each period as the denominator. Cumulative incidences per 10,000 population were mapped at the community level and at the district level using ArcGIS 9.3.1 [26]. The methodology for mapping at the district level is described in Text S1. The average incidence per 10,000 population was calculated to investigate the global clustering among communities. Smoothed risk estimates were calculated for each five-year time period using the Empirical Bayes Smoothing (EBS) in the GeoDa software package [27]. The EBS technique can be used to adjust for instability in the risk estimates caused by heterogeneity in the distribution of cases and the population. Posterior risk is estimated from a weighted combination of the local risk and the risk in the surrounding areas (the prior). It has been suggested that the EBS methodology can be implemented in several scenarios, such as when the numerator data total less than three cases, which was the situation in this analysis [28]. In order to maintain a standard comparison of rates between time periods, crude and EBS estimates of cumulative incidence rates (per 10,000) were mapped using graduated symbols with the same data bins (0–3, 4–9, 10–18, 19–30, 31–50, >50). A chi-square analysis was used to test for a relationship between the number of communities reporting anthrax in the east and west of the country during each time period. Boxplots were used to illustrate differences between the crude and EBS rates for both of the five-year time periods.

### Spatial Autocorrelation Analysis

To test for spatial dependence in EBS average anthrax incidence rates between communities, the global Moran's *I* test was implemented using OpenGeoDa [29]. This test analyzes the degree of spatial autocorrelation present in the data in relation to a single variable of interest and is written following Moran [30]:
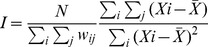
where *N* is the number of communities, 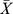 is the average EBS incidence at each community, *X*_i_ and *X*_j_ are the EBS incidence in community *i* and *j*, and *W*_ij_ is the spatial weight matrix defining the spatial relationships between communities. In this instance *W*_ij_ was defined as a series of increasing distances from 1 to 10 kilometers (km) to test a range of dependencies. The statistic produces values between −1 and +1 similar in interpretation to the Pearson's correlation with large positive values of *I* indicating the similarity of values between locations with the presence of either positive or negative autocorrelation and negative values indicating a dissimilarity among values. Significance of the test statistic was assessed with a pseudo p-value generated using 999 random permutations [29].

### Spatial and Space-Time Cluster Analysis

To identify the spatial and spatio-temporal clustering of anthrax cases during the 10-year period the Poisson clustering models using the space-only and space-time scan statistics were implemented in SaTScan v9.0 [31]. SaTScan uses a series of moving windows of varying diameter to detect spatial clusters across a study area and simultaneously implements a series of cylinders of varying height to detect temporal clustering as well [29]. The relative risk (RR) is calculated based on the number of observed and expected observations and the likelihood function for each window location and size depending on the assumed distribution [32]. For the SaTScan analyses, community latitude and longitude coordinates were used as the case locations. The model was run on yearly case data, using the total number of yearly cases per community while adjusting for the underlying population of each community. Space-only models were run for each five-year time period using 25% of the population at risk. This threshold for the population at risk was chosen to identify local clusters of anthrax rather than using the default of 50%. Maximum spatial extents of 25%, 20%, 15%, and 10% of the population at risk were chosen with no change in cluster size or location, therefore 25% was used in the analysis. All SaTScan cluster were chosen at the p≤0.05 level of significance.

In this study we were particularly interested in identifying areas of human anthrax that experienced recurrent infections or persistence over space and time. To identify persistence we implemented the Space-Time Poisson model in SaTScan using 25% of population at risk with a maximum temporal window of 90% of the study period. The temporal window was extended to 90% to allow SaTScan to search across a greater portion of the study period and thereby identify communities that may have persisted as clusters in both space and time across a large temporal window.

Additionally, a SaTScan model examining spatial variation in a temporal trend was used to identify individual communities that may have been contributing to an increasing trend in incidence over time. SaTScan fixes the temporal window during the analysis and compares the trend in risk inside and outside of the varying size spatial window [32], [33]. The most likely spatial cluster location is chosen by the cylinder that maximizes the change in the trend over time.

### Environmental and Anthropogenic Variables

Environmental and anthropogenic variables used in this analysis have been suggested in the literature to be associated with the presence of human anthrax (Table 1) [32], [33]. Continuous environmental variables included elevation, average annual precipitation [32], mean land surface temperature (LST), mean mid infra-red reflectance (MIR) [3], [6], [7], [8], and minimum soil pH (soil pH) [34]. Continuous anthropogenic variables included population density [35], travel time to cities with a population >50,000 (travel time) [36] and categorical variables for urban/rural classification (UR) [26] and cattle density dichotomized into equal groups <19 head per 1 km^2^ and >19 head per 1 km^2^ (cattle density) [37].

**Table 1:** Environmental and anthropogenic variables used in the logistic regression model.

Environmental Variables	Rationale
Mean Annual Precipitation	Precipitation has been shown to be associated with the distribution of the bacterium and seasonal outbreak events.
Elevation	Low lying elevations may act as storage areas for the pathogen as a result of surface runoff from storms.
Mean Mid Infra-Red Reflectance	Soil moisture is suggested to be associated with the presence of the bacterium.
Mean Land Surface Temperature	The bacterium prefers a temperature range between 18° and 39°C.
Minimum Soil pH	Alkaline soils with a pH>6.0 are considered favorable for the pathogen.
**Anthropogenic Variables**	
Cattle Density	Areas with higher cattle density would likely have more agriculture and a greater chance of coming into contact with infected animals.
Urban/Rural	Rural areas often experience a higher burden of disease due to the frequency of peridomestic living arrangements.
Travel Time to City >50,000	Longer travel times are more indicative of rural areas and higher agricultural intensity.
Population Density	Lower population density may be associated with rural areas and agricultural production.

### Logistic Regression

Logistic regression was used to identify differences in risk factors between communities that were identified as a persistent space-time cluster in SaTScan and those communities that were not. A total of nine environmental/anthropogenic variables (Table 1) were used to construct univariate, and a final multivariate, logistic regression models using a binary response 0/1 with clustered communities = 1 (*n* = 24) and non-clustered communities = 0 (*n* = 80) as the dependent variable. The final model was constructed in SAS using a backward stepwise approach with entry p-values of 0.15 and stay p-values of 0.20. Variables used in the stepwise model selection were limited to those that did not show significant collinearity (ρ_s_≤0.70) using a Spearman's rank correlation test. Goodness of fit was evaluated with the Hosmer and Lemeshow test and interaction terms were tested between categorical and continuous independent variables. To assess the discrimination ability of the final logistic regression model we used receiver operating (ROC) curves, based on the area under the curve (AUC) [38]. The AUC provides a measure of model accuracy with values ranging from 0.0 to 1, where values of 1 indicates all locations were correctly classified by the model and 0.5 indicating no better than random.

## Results

### National Incidence

**Figure 1:**
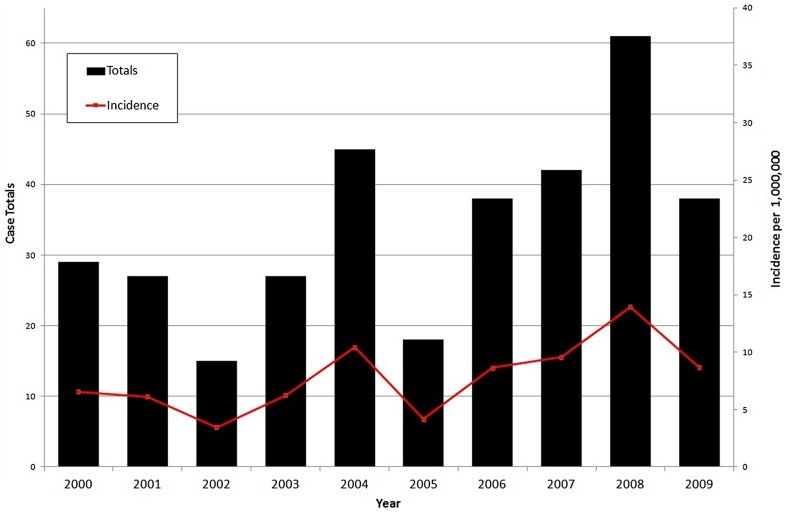
National reporting of human cutaneous anthrax in Georgia by year and incidence per 1 million population.

During the study period there were 340 cases of human anthrax reported in Georgia with a median number of reported cases per year of approximately 33.5 (CI_95%_: 22.5, 42.0). Yearly national incidences per one million displayed inter annual variability ranging from a low of 3.4 (CI_95%_:1.9, 5.7) in 2002 and to a high of 13.9 (CI_95%_: 10.7, 17.9) in 2008 (Figure 1). There were 143 cases [range: 15–45] during period one (2000 to 2004) with a median number of yearly cases of 27 (CI_95%_: 9, 39) and a total of 197 cases [range: 18–61] during period two (2005 to 2009) for a yearly median of 38 (CI_95%_: 15, 58).

### Community Level Analysis

**Figure 2:**
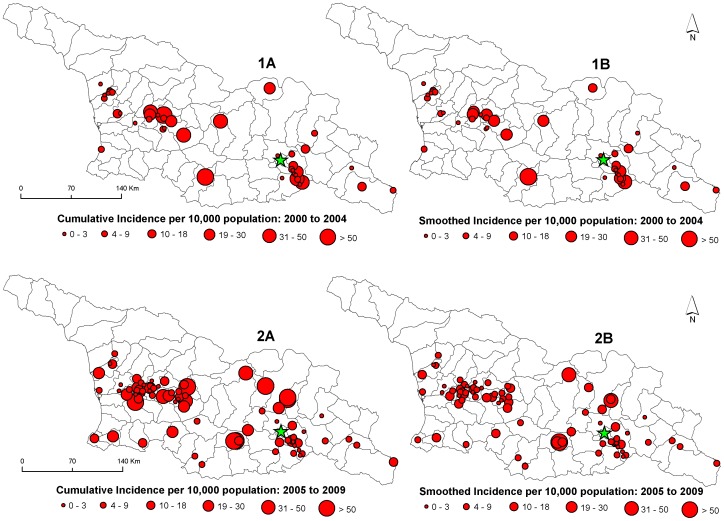
Maps showing the cumulative incidences of human cutaneous anthrax in Georgia at the community level. Symbol sizes represent cumulative incidence risk per 10,000 population for period 1 (1A) and period 2 (2A). Empirical Bayes Smoothing estimates were calculated for both period 1 (1B) and period 2 (2B) to adjust for population heterogeneity.

Figure 2 shows the spatial distribution of crude (insets 1A and 2A) and smoothed (insets 1B and 2B) community level cumulative incidence rates in Georgia for each time period, respectively. There were a greater number of communities reporting human anthrax during period two (*n* = 79) compared to period one (*n* = 44) with an increased number occurring in the west of the country during period two (*n* = 47) compared to period one (*n* = 23); however this difference was not significant (χ^2^ = 0.6, p = 0.44). . Community level cumulative incidence rates per 10,000 population during period one ranged from a low of 0.09 (CI_95%_: 0.05, 0.17) to a high of 136.4 (CI_95%_: 83.3, 210.07) and during period two ranged from a low of 0.18 (CI_95%_: 0.11, 0.29) to a high of 242.3 (CI_95%_: 97.07, 499.2). Box plots illustrated that the cumulative incidence rates for each time period were unstable due to population heterogeneity with a greater than 30% reduction in the estimates applying an EBS rate adjustment (Figure S2). District level estimates of cumulative risk are displayed in Figure S3.

### Cluster Analysis

**Figure 3:**
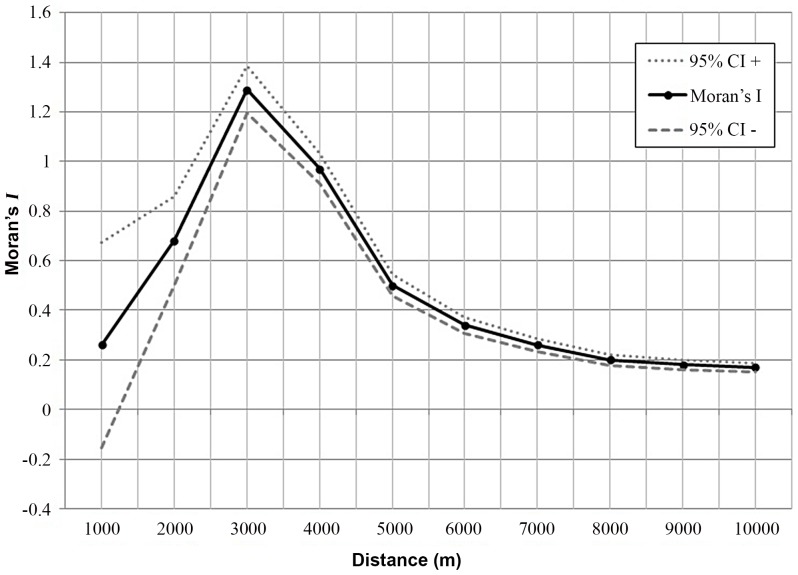
Spatial correlogram displaying global Moran's *I* values at varying distance thresholds. Distances ranged from 1,000 m to 10,000 m by 1,000 m increments. Dashed lines represent 95% confidence intervals for the Moran's *I* values.

**Figure 4:**
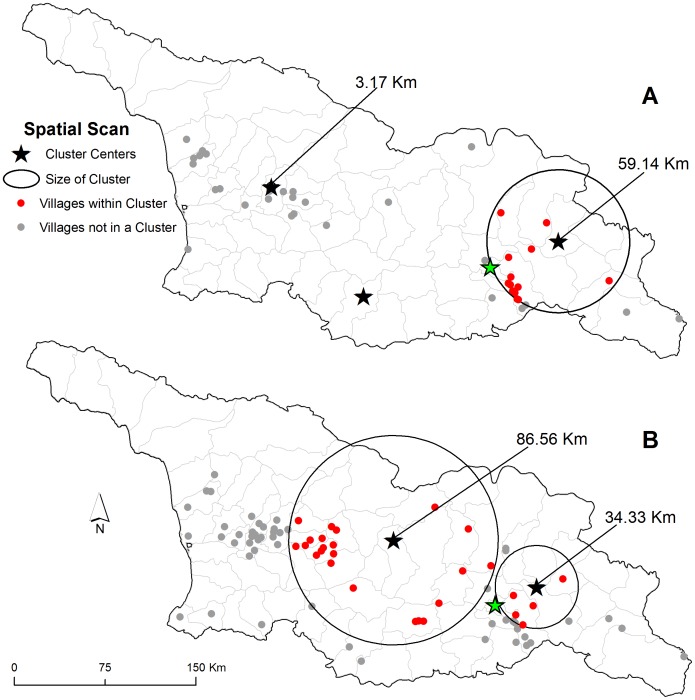
Spatial clustering of human cutaneous anthrax. Village level clustering was identified during the period 2000 to 2004 (A) and 2005 to 2009 (B). Star symbols represent cluster centers, red dots represent villages that were part of spatial cluster, and grey dots represent villages that were not part of a spatial cluster. Buffers of clusters are represented in kilometers (km).

The Moran's *I* test identified the presence of significant positive spatial autocorrelation (Figure 3). A correlogram of Moran's *I* index values showed increases in positive values up to a distance of 3000 meters and then a subsequent sharp decline up to a distance of 8000 meters indicating spatial dependence at a relatively local scale. The space-only SaTScan analysis detected the presence of three significant clusters during period one (Figure 4A) and two clusters during the period two (Figure 4B). Spatial clusters in period one were smaller, and contained fewer communities compared to period two. Clusters varied spatially between periods with a large cluster in period two located in the center of the country that was absent during period one. Persistent clusters in both periods were identified around the main urban center, the capital Tbilisi in the southeast.

**Figure 5:**
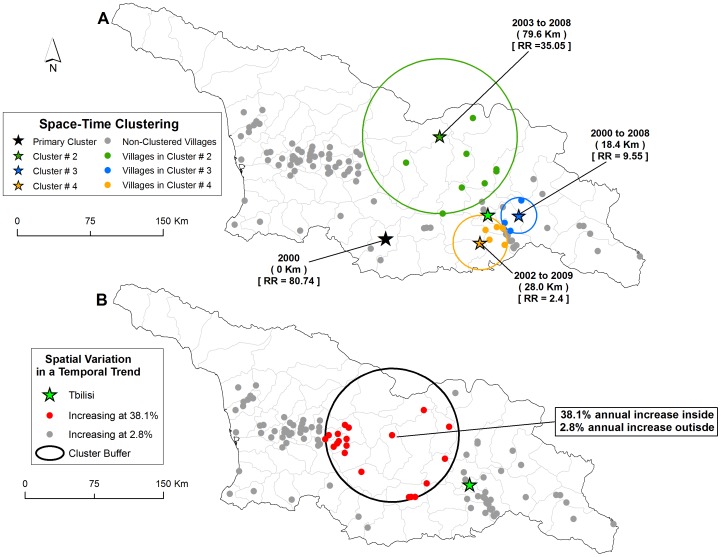
Distribution of space-time clusters defined as persistence and the spatial variation in a temporal trend model. Star symbols in (map inset A) represent the center of clusters defined as persistence, colored dots correspond to villages that were included within each specific space-time clusters, grey dots represent villages not part of a cluster, relative risk for each cluster is displayed (RR) along with the buffer size in kilometers (Km). Red dots in inset B refer to villages within the buffer that are increasing at a faster rate (38.1% annually) compared to those outside the buffer shown in grey dot (2.8% annually).

Results from the space-time model identifying persistence revealed the presence of four significant persistent clusters with two cluster located near the capital Tbilisi (Figure 5A). Clustered communities had an average of 1.01 cases per year compared to 0.2 cases per community outside of clusters. Clusters were predominantly located in the central and eastern part of the country excluding a large number of communities in the west. The spatial variation in a temporal trend model indicated a single cluster of communities that were contributing to an increasing trend in human anthrax reporting (Figure 5B). Communities within the central part of the country were shown to have incidence of anthrax increasing at an average of 38.1% per year as compared to 2.8% per year for the rest of the communities in the study area.

### Logistic Regression Analysis

Results from the logistic regression models are shown in Table 2. The Hosmer and Lemeshow test (*p* = 0.96) revealed the model was a good fit to the data. There were no statistically significant interaction terms at the p≤0.10. Accuracy of the final main effects model evaluated by the AUC from the ROC analysis showed good discrimination (AUC = 0.85, *p*<0.001).. Environmental and anthropogenic factors were both found to be associated with the presence of persistence. Strong positive associations shown in odds ratios (OR) were found between soil pH (OR 4.58, CI_95%_: 1.55, 13.51), and UR (urban areas have greater odds OR, 4.67 CI_95%_: 1.11, 19.64). Cattle density (cattle density above 19 head per 1 km^2^ have greater odds, OR 2.78, CI_95%_: 0.91, 9.55) was also shown to be positively associated although not at the 0.05 level of significance (*p* = 0.10).

**Table 2:** Results of logistic regression examining environmental and anthropogenic factors associated with human anthrax clusters.

Variables*	Full Model (OR)	95% CI	Final Model (OR)	95% CI	*χ* ^2^ (p-value)
Mean Annual Precipitation	1.00	0.99, 1.01	**-**	**-**	
Elevation	1.00	0.99, 1.00	**-**	**-**	
Mean Mid Infra-Red Reflectance	1.00	0.96, 1.05	1.04	1.01, 1.08	0.03
Mean Land Surface Temperature	0.89	0.50, 1.58	0.65	0.40, 0.82	<0.01
Minimum Soil pH	3.31	0.89, 12.34	4.58	1.55, 13.51	<0.01
Cattle Density <19 (Ref)	1.00	-	1.00	-	0.10
>19	2.41	0.62, 9.44	2.78	0.91, 9.55	
Rural (Ref)	1.00	-	1.00	-	0.03
Urban	4.00	0.65, 25.16	4.67	1.11, 19.64	
Population Density	1.00	0.99, 1.00	**-**	**-**	
Travel Time	1.00	1.00, 1.01	**-**	**-**	

* Results of the models are reported in odds ratios (OR) with corresponding 95% confidence intervals (CI).

## Discussion

Anthrax in the country of Georgia continues to represent a threat to public and veterinary health. The current national classification of endemicity does not allow for an efficient implementation of control measures given the limited resources available locally. Previous human studies have focused on national reporting [39], investigations of a single outbreak, or outbreaks across a short period of time [40]. In contrast, this study represents a fine scale spatial investigation of naturally acquired human anthrax during a decade of reporting.. We identified persistent clusters of disease at the community level that were associated with both environmental and anthropogenic factors. Establishing these sub-national estimates of human anthrax risk provides a crucial first step in implementing targeted public health interventions.

The high rates of anthrax observed during the study period are likely due to a combination of factors resulting from decreased funding for public and veterinary health management following the collapse of the Soviet Union as well as changes to agricultural production [17]. Since the transition to independence in the 1990s, reliance on agriculture has grown with an increase in agricultural employment from 25% in 1990 to ∼55% in 2009 [18], [41], [42]. During this period agricultural decollectivization has marked a switch to individual ownership [5], [43]. This has resulted in a greater number of individual animal holdings and a subsequent increase in peridomestic arrangements [43]. Greater contact among livestock and humans living in close proximity (peridomesticity) has been shown elsewhere to facilitate disease transmission [44]. The increases in reporting observed here may have been due to increased diagnostic capacity and awareness, although the issue of anthrax as a neglected, endemic disease [43], along with funding cuts to public health facilities, has likely led to an under reporting in both humans and animals [45]. In Georgia the number of documented livestock cases are few, with the number of human cases out of proportion suggesting an anthropocentric reporting system [3]. Additionally, the absence of gastrointestinal cases of anthrax during the study period, given the large number of cutaneous cases, further highlights the probable under reporting of the disease [5].

Results from this study point to a primarily localized transmission of human anthrax identified by the strong spatial dependence of the Moran's *I* statistic at a relatively short distance (3000 meters) (Figure 3). This is consistent with research that has described the local dynamics of infection related to the communal handling, butchering, or sharing of infected meat across short distances [14], although imported cases from contaminated animal products have been documented elsewhere [46]. In Georgia, anthrax was spatially heterogeneous at the community level as evident by the presence of clustering (Figure 4). These findings are similar to studies in livestock/wildlife, which are often the source of human infection, that found variation in cases related to environmental constraints on the pathogen [42], [47], [48]. Reports on the spatial distribution of human cases are largely absent in the literature although in Kazakhstan human reporting was shown to be located in specific areas that comprised historical foci of the disease [49]. By expanding the maximum temporal window to 90% in the SaTScan space-time analysis we identified areas that represented anthrax persistence over time. The possibility persistence is likely due to a combination of ecological factors that permit pathogen survival and anthropogenic factors that accommodate disease transmission [20], [48], [50].

Soil pH showed a strong positive association with anthrax persistence, as documented elsewhere [51]. Additional environmental factors MIR and LST were also significant in the model. Alkaline soils with adequate moisture (MIR) and an ambient temperature above 15°C (LST) have been suggested as factors related to incubator areas for bacterium [8], [52], [53]. These factors are similar to previous studies that have modeled the potential distribution *B. anthracis* in the United States and Kazakhstan [7], [8], [50]. Soil pH has long been considered an important factor for spore survival with the bacterium preferring alkaline soils with a relatively high pH [7]. In Tanzania, a higher proportion of anthrax seropositive wildlife and domestic animals were identifed in areas with alkaline soils [8], [10]. Arable land is limited in Georgia (∼17% of all land), leaving pasture/grasslands for livestock grazing in more alkaline soils [7], [54], [55]. Cattle density had a positive association OR 2.78 (CI_95%_: 0.91, 9.55) with persistence, however, it was not significant. Infection ratios comparing humans to livestock can vary widely, ranging from 1∶3 to 1∶16 as seen in Zambia [50] and the density of cattle may not be as important as the presence or absence of livestock.

Human anthrax has been described as a disease of rural communities [43]. However, our study suggests that urban areas had >4.5 times the odds of being a persistent cluster when compared to rural areas. Health seeking behaviors of urban and rural populations differ, which can bias reporting due to access to healthcare facilities. In the Caucasus region, rural populations were shown to under-utilize the national healthcare leading to an under reporting of illnesses [47]. Anthrax infections in livestock, as seen elsewhere may also prompt owners to slaughter animals and bring the meat to market more quickly [6]. Urban areas often function as the market centers, hence contaminated livestock brought to market may increase the risk of exposure. Also, occupational exposures may differ in and around urban areas with animal processing facilities close to central markets.

This study had several limitations that warrant discussion. The study was an ecological analysis that examined aggregate level data and may not represent the true associations of risk. Deriving census population as denominator data for small populations is often difficult, particularly in low resource settings. In this study, population totals for most communities were obtained from census records although several estimates were also obtained from gridded population data, which may skew the cumulative risk estimates and SaTScan results by either overestimating or underestimating the denominator figures. Model accuracy of the logistic regression was not evaluated with training and testing data due to the sample size of the persistent clusters identified. Although the disease was likely under reported, the reporting may have been biased towards urban areas with more access to healthcare facilities resulting in an under estimation of risk in rural areas. Furthermore, the method of classifying urban areas, while well documented in the literature, may in some instances incorrectly classify communities. Epidemiological data were not available on individuals, therefore individual level risk factors such as age, gender, and the source of infection were not considered in this study. Although anthrax is associated with specific factors on the landscape identified here, cultural and socio-economic factors not available in this study may also influence the occurrence of the disease.

Research has reiterated the need for continued surveillance in order to distinguish between an intentional release and natural occurring disease [56]. This is especially true in light of the 2001 anthrax bioterrorist attack in the United States, which has renewed fears of its use as a weaponized biological agent [18]. Public health officials may be able to implement directed intervention strategies targeting high risk areas identified in this study which can include: training on proper carcass disposal, targeted livestock vaccination programs, education about the disease, and limiting occupational exposures through personal protective equipment [26], [57], [58]. While changing behavior related to zoonotic disease transmission may be difficult [26], [59], initiating targeted livestock vaccination in areas with persistence risk can help to control future outbreaks. Future research should focus on collecting epidemiologic data on individual cases while also further exploring factors associated with an increasing rate of disease in communities identified in this study.

## Supporting Information

Figure S1 **Average incidence per 10,000 population of human cutaneous anthrax at the community within Georgia during the period 2000 to 2009.** Cattle density is shown in two categories with areas in green representing ≥19 head of cattle per square kilometer and lighter colored areas representing.
(PDF)

Figure S2 **Box plots showing crude and Empirical Bayes smoothed (EBS) estimates for cumulative incidence for the period 2000 to 2004 and 2005 to 2009.**
(PDF)

Figure S3 **Smoothed cumulative incidence for each 5 year period (top and middle) and the percent difference between the two periods (bottom) at the rayon level.**
(PDF)

Text S1 **Methodology for district level mapping.**
(PDF)
