# Supplementary figures and images for: Evidence of Local Persistence of Human Anthrax in the Country of Georgia Associated with Environmental and Anthropogenic Factors
Source: PLoS Negl Trop Dis. 2013 Sep 5;7(9):e2388. doi: 10.1371/journal.pntd.0002388 (PMC3764226; doi:10.1371/journal.pntd.0002388)

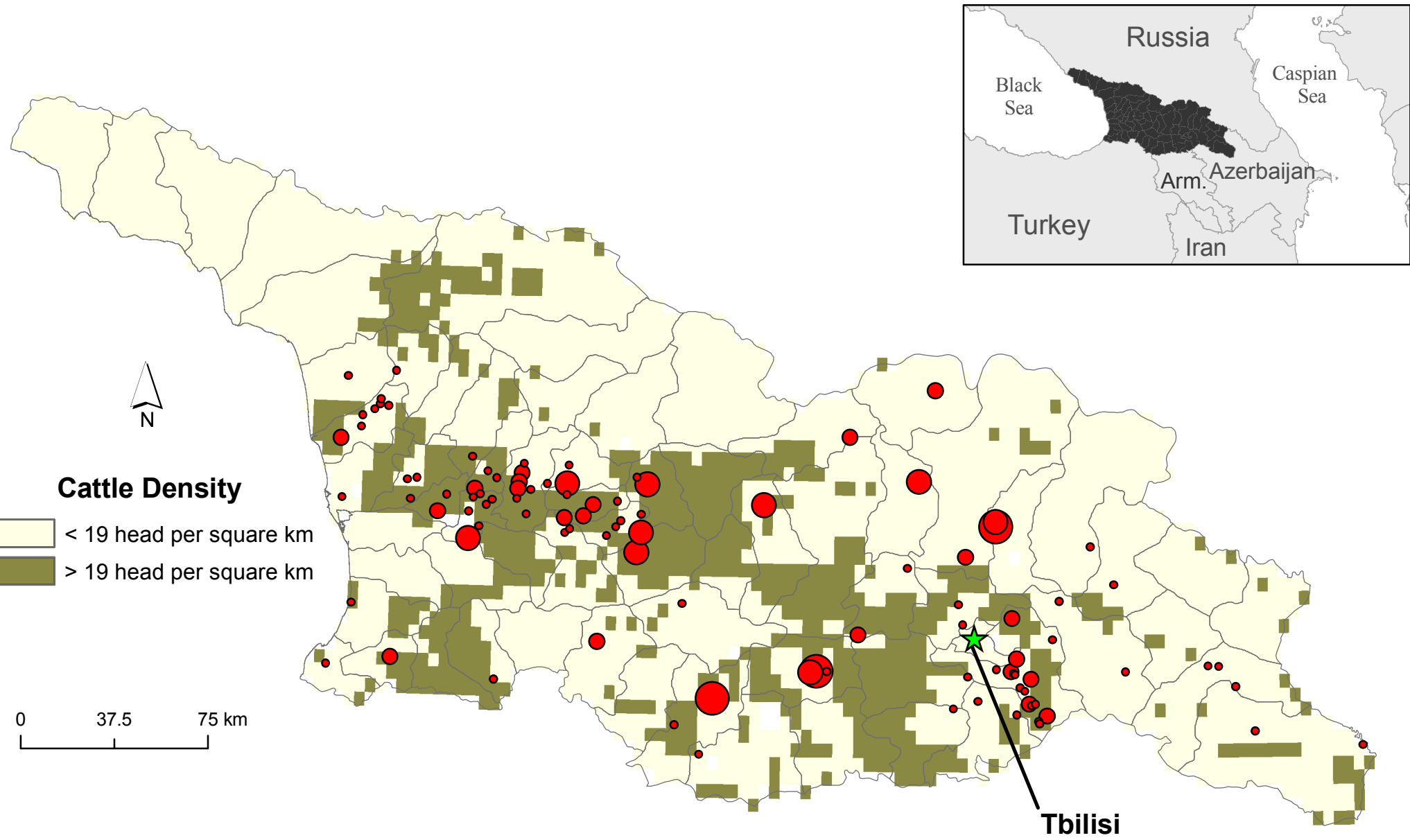

Supplement: Figure S1 — Average incidence per 10,000 population of human cutaneous anthrax at the community within Georgia during the period 2000 to 2009. Cattle density is shown in two categories with areas in green representing ≥19 head of cattle per square kilometer and lighter colored areas representing. (PDF) [file pntd.0002388.s001.pdf]

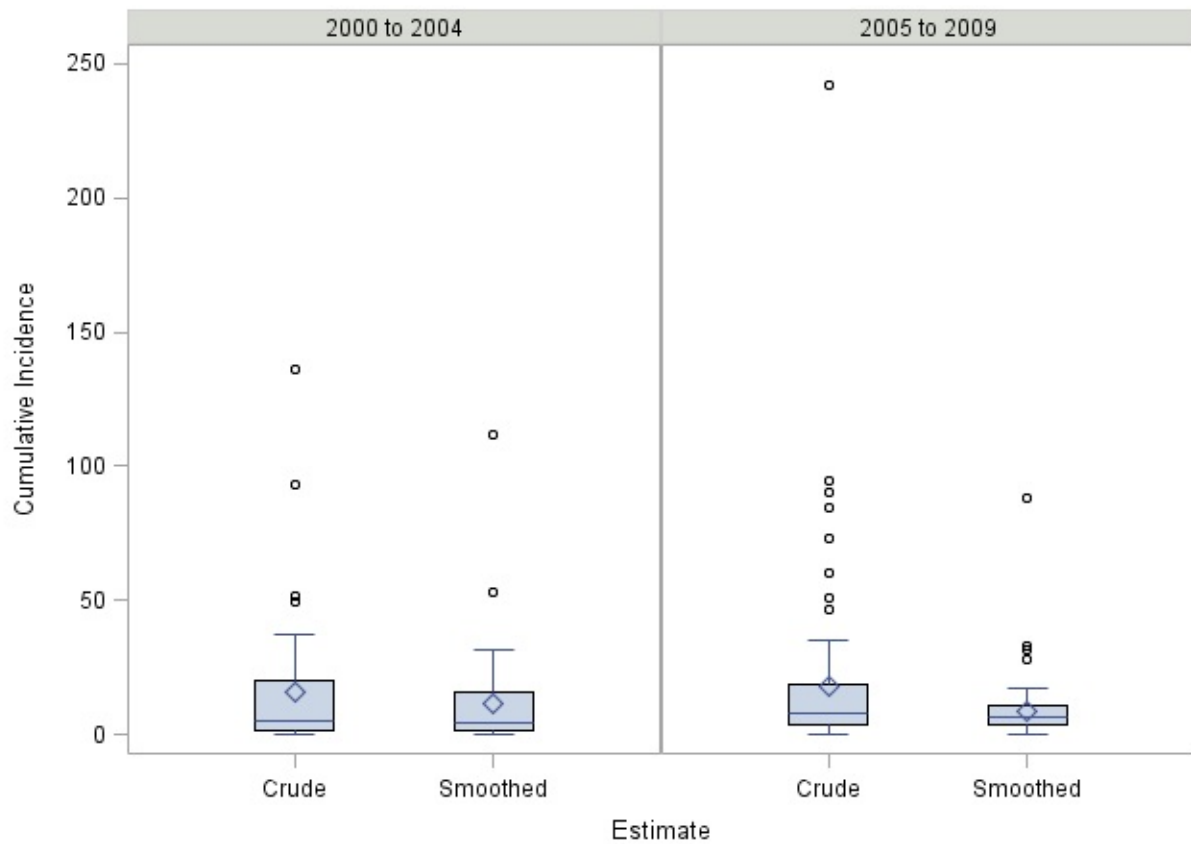

Supplement: Figure S2 — Box plots showing crude and Empirical Bayes smoothed (EBS) estimates for cumulative incidence for the period 2000 to 2004 and 2005 to 2009. (PDF) [file pntd.0002388.s002.pdf]

### Human Anthrax 2000 - 2004

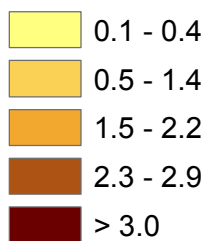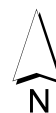

0 25 50 100 Kilometers

### Human Anthrax 2005 - 2009

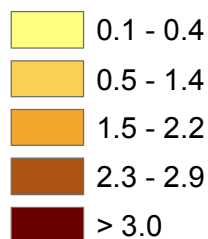

### Percent Change

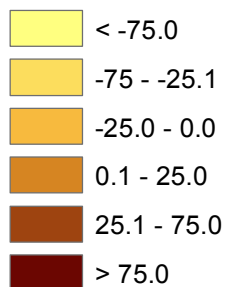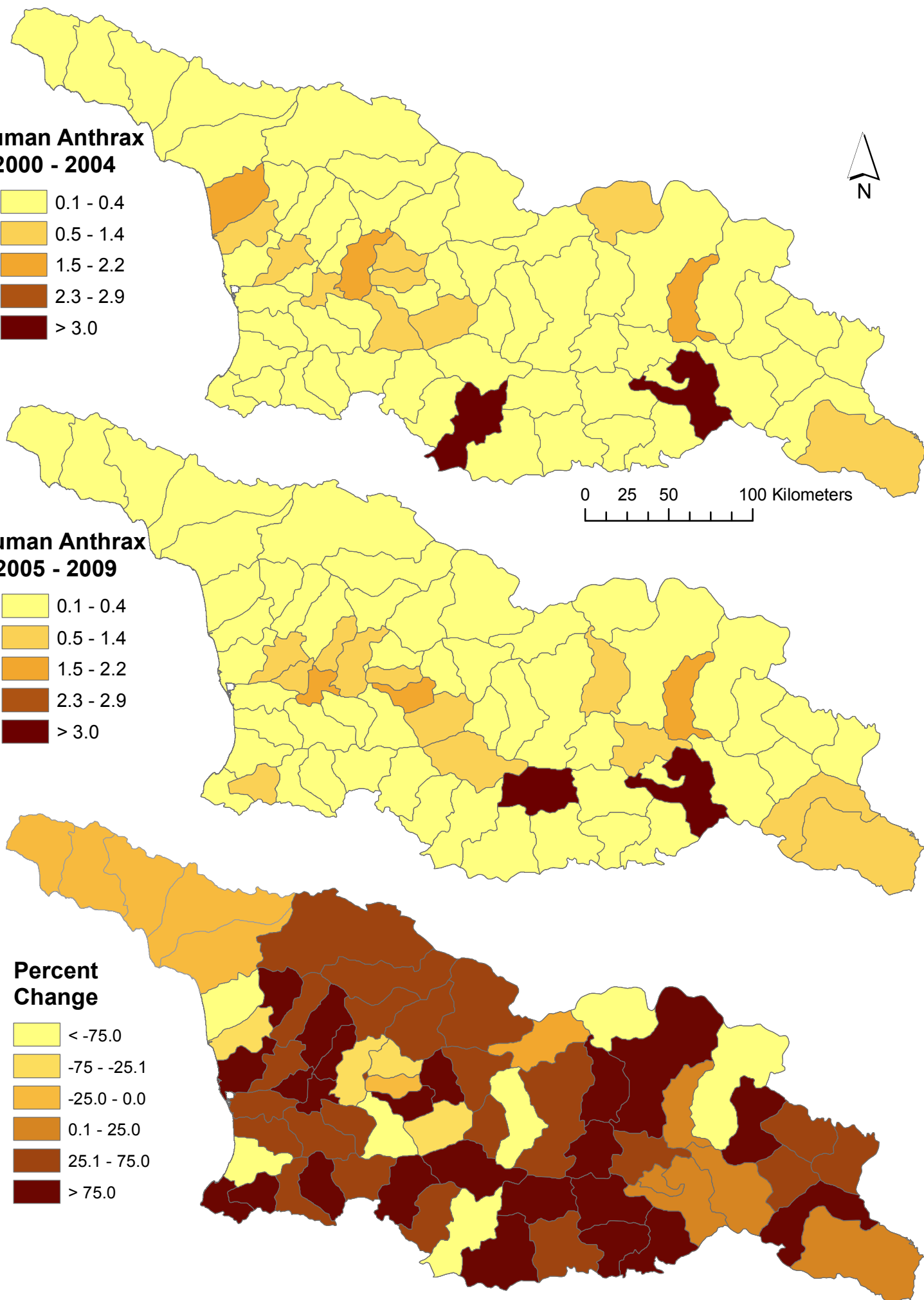

Supplement: Figure S3 — Smoothed cumulative incidence for each 5 year period (top and middle) and the percent difference between the two periods (bottom) at the rayon level. (PDF) [file pntd.0002388.s003.pdf]
