# Supplementary material for: Evidence of Local Persistence of Human Anthrax in the Country of Georgia Associated with Environmental and Anthropogenic Factors
Source: PLoS Negl Trop Dis. 2013 Sep 5;7(9):e2388. doi: 10.1371/journal.pntd.0002388 (PMC3764226; doi:10.1371/journal.pntd.0002388)
Supplement: Text S1 — Methodology for district level mapping. (PDF) [file pntd.0002388.s004.pdf]

To capture changes in rates of human cutaneous anthrax reporting between the two time periods 2000-2004 and 2005-2009, data from the community level were aggregated to the second administrative boundary (district level) using a shapefile obtained from the Global Administrative boundaries database (http://www.gadm.org/). This was done in order to maintain a standard of comparison between the two time periods since reporting among villages varied during the study period. In this manner a village reporting cases in period 1 and not period 2 (and vice versa) would not contribute to a large percent change in the incidence of human anthrax if other communities in that district reported cases. The cumulative incidence per 10,000 population for each district was calculated using the total number of cases reported during each time period divided by the median year population of each time period obtained from the National Statistics Office of Georgia (http://www.geostat.ge/). Smoothed risk estimates were calculated for each five-year time period using the Empirical Bayes Smoothing (EBS) in the GeoDa software package [1]. The EBS technique can be used to adjust for instability in the risk estimates caused by heterogeneity in the distribution of cases and the population. Posterior risk is estimated from a weighted combination of the local risk and the risk in the surrounding areas (the prior). It has been suggested that the EBS methodology can be implemented in several scenarios, such as when the numerator data total less than three cases, which was the situation in this analysis [2]. In order to maintain a standard comparison of rates between time periods EBS estimates of cumulative incidence rates (per 10,000) were mapped using graduated symbols with the same data bins (0.1-0.4, 0.5-1.4, 1.5-2.2, 2.3-2.9, >3.0).

The percent change in reporting between the two time periods was calculated using the following formula: [Cumulative Risk in Time Period 2 - Cumulative Risk in Time Period 1]/ Cumulative Risk in Time Period 1. All data were mapped using ArcGIS 9.3.1 [3].

1. Anselin L, Syabri I, Kho Y (2006) GeoDa: An introduction to spatial data analysis. Geographical Analysis 38: 5-22.
2. Waller LA, Gotway CA (2004) Applied spatial statistics for public health data: Wiley-Interscience.
3. ESRI A (2009) 9.3. 1. Environmental Systems Research Institute, Redlands, United States.
